# Retraction: Redundant Publication of the article Dental caries and oral health practices among 12 year old children in Nairobi West and Mathira West Districts, Kenya. Gladwell Gathecha et al. The Pan African Medical Journal. 2012;12:42

**DOI:** 10.11604/pamj.2015.22.233.8358

**Published:** 2015-11-11

**Affiliations:** 1 The Pan African Medical Journal, Yaoundé, Cameroun

**Keywords:** Retraction, scientific misconduct, duplicated publication

## Abstract

This retracts article Dental caries and oral health practices among 12 year old children in Nairobi West and Mathira West Districts, Kenya. Gladwell Gathecha, AnselimoMakokha, Peter Wanzala, Jared Omolo, Perry Smith. The Pan African Medical Journal. 2013;12:42.

## Retraction

We hereby inform to our readership of the retraction of the article *Dental caries and oral health practices among 12 years old children in Nairobi West and Mathira West Districts, Kenya. Gladwell Gathecha, Anselimo Makokha, Peter Wanzala, Jared Omolo, Perry Smith*. From our investigation, it clearly appears that the article was published in the ***Journal of the Kenya Dental Association*** in January 2011 [1]. The article was then submitted to the Pan African Medical Journal on 6 September 2011 [2]. Therefore, we withdraw this article of the medical literature in accordance with the guidelines and best editorial practices of the Committee on Publication Ethics. We apologize to our audience for this unfortunate situation.
